# Observational study of long-term persistent elevation of neurodegeneration markers after cardiac surgery

**DOI:** 10.1038/s41598-019-42351-2

**Published:** 2019-05-09

**Authors:** Matthew DiMeglio, William Furey, Jihane Hajj, Jordan Lindekens, Saumil Patel, Michael Acker, Joseph Bavaria, Wilson Y. Szeto, Pavan Atluri, Margalit Haber, Ramon Diaz-Arrastia, Krzysztof Laudanski

**Affiliations:** 1 DO/MBA Student, Philadelphia College of Osteopathic Medicine, Philadelphia, Pennsylvania USA; 2 Department of Nursing, Widener University, Chester, Pennsylvania USA; 3 Department of Nursing, University of Pennsylvania, Philadelphia, Pennsylvania USA; 4 Department of Anesthesiology and Critical Care, Hospital of University of Pennsylvania, Philadelphia, Pennsylvania USA; 5 Department of Cardiovascular Surgery, Hospital of University of Pennsylvania, Philadelphia, Pennsylvania USA; 6 Department of Neurology, Penn Presbyterian Medical Center, Philadelphia, Pennsylvania USA

**Keywords:** Biomarkers, Molecular medicine

## Abstract

Surgery and anesthesia induce inflammatory changes in the central nervous system, which ultimately lead to neuronal damage concomitant with an increase in the level of neurodegeneration markers. Despite some experimental data showing prolonged activation of the immune system post-surgery, no study has determined the extent of long-term elevation of neurodegeneration markers. The purpose of this study was to investigate the serum levels of tau protein, ubiquitin carboxyl-terminal hydrolase L1 (UCH-L1), neurofilament light (NF-L), and glial fibrillary acidic protein (GFAP) after elective cardiac surgery with the implementation of cardiopulmonary bypass (CPB). The serum levels of these markers from 30 patients were compared longitudinally to the baseline (pre-surgery or t_0_), at 24 hours (t_+24_), at 7 days (t_+7d_), and at 3 months (t_+3m_). The secondary outcome was the production of macrophage-colony stimulating factor (M-CSF) and tumor necrosis factor-α (TNF-α) *in vitro* by isolated monocytes in response to lipopolysaccharide (LPS) as the measure of immune system activation. The tertiary outcome was the serum level of C-reactive protein (CRP), serum amyloid P (SAP), and α-2-macroglobulin (A2M). Serum levels of tau protein increased 24 hours after surgery (p = 0.0015) and remained elevated at 7 days (*p* = 0.0017) and three months (*p* = 0.036). Serum levels of UCH-L1 peaked at 24 hours (*p* = 0.00055) and normalized at 3 months. *In vitro* secretion of M-CSF by LPS-stimulated peripheral monocytes, but not TNFα, correlated highly (*r* = 0.58; *p* = 0.04) with persistent elevation of serum tau levels at 3 months. The serum CRP and SAP increases correlated with tau post-CPB levels significantly at 3 months. We demonstrated that elevation of serum tau levels at 24 hours, 7 days, and 3 months after heart surgery is concomitant with some traits of inflammation after CPB. The elevation of tau several weeks into recovery is significantly longer than expected.

## Introduction

There is well-established evidence suggesting that surgery and anesthesia have a significant impact on immediate neurocognitive recovery^1–5^. However, the connection between long-term cognitive functioning and the emergence of neurodegenerative disorders such as Alzheimer’s or Parkinson’s disease, and a para-surgical insult preempting the emergence of symptoms by a long period of time, has been debated^2,4^. This is of particular importance in elderly subjects^6–11^. The effect of para-surgical stress can amplify the detrimental effects of common elderly comorbidities such as malnutrition, deconditioning, and delirium^4,10^.

The exact mechanism of postoperative cognitive decline is not clear but exceeding the ability of allostatic coping to para-surgical stress will result in a new immunostasis^9^. In particular, the immune system may evolve towards prolonged subclinical activation^12^. A rise in neurodegeneration markers has been noted in cerebrospinal fluid after cardiac surgery both with and without the implementation of cardiopulmonary bypass (CBP)^13^. Most studies focused on a short-term observation window not exceeding 72–96 hours. The development of ultra-sensitive techniques (SiMoA™, single molecule arrays, single molecule enzymatic assay) has enabled robust measurements of markers in blood. Blennow *et al*. (2011) showed that anesthesia and/or surgery result in a temporary increase of tau protein and neurofilament light in the serum up to 96 hours after non-cardiac surgery^14^.

The underlying pathogenesis of cognitive dysfunction after surgery is debated and multifaced. The relationship between microemboli and cognitive dysfunction has been discussed^15,16^. Abnormalities in arterial and venous flow were suggested as well^17^. Some emphasized the effect of hypotension/hypoxia, hypercarbia, and anesthetic toxicity as contributing factors. However, neuroinflammation occupies the most dominant place in a discussion of postoperative cognitive decline^3,11,18^. Post-cardiac surgery inflammation leads to an elevation in free radicals, acute phase proteins, complement abnormalities, and cellular abnormalities^3,18,19^. The lack of correlation with the degree of surgical injury and postoperative decline suggests local inflammatory mechanisms^16,20,21^. Activation of native resident and brain-specific macrophages is often implicated as the driving force behind post-cardiac surgery neuronal damage^19,22^. Importantly, once activated, microglia can remain active for a prolonged time after surgery or any priming event^22–24^. Production of M-CSF is characteristic of atypically activated monocytes and microglia and has been linked to neurodegeneration^22,25–28^. Several functions and regulation characteristics of microglia are mimicked by peripheral blood monocytes (MO)^27,29^. Considering that obtaining tissue samples from a living donor is prohibitive, monitoring the function of blood MO allows for an estimation of the neuroinflammatory process^23,30^.

In this study, we investigated the dynamics of four markers of neurological injury after heart surgery involving cardiopulmonary bypass. Tau protein is a well-known marker associated with Alzheimer’s disease (AD)^4,30,31^. Ubiquitin carboxyl-terminal hydrolase L1 (UCH-L1) is neuron-specific and required for normal synaptic and cognitive function^32^. UCH-L1 gene is linked to AD, traumatic brain injury, PD, and an increase in extracellular fluid suggesting neuronal injury^33^. Neurofilament light (NF-L) is emerging as a serum marker of axonal neurodegeneration in AD and other neurodegenerative disorders, closely tracking the degree of tissue damage^34,35^. Glial fibrillary acidic protein (GFAP) is produced by glial cells and astrocytes after PAMP stimulation leading to neuronal tissue protection, but when apoptosis occurs GFAP leaks into CSF and blood^36–39^. Considering that neuroinflammation has been shown to be much more persistent after surgery, we hypothesized that markers of neuronal damage may persist as long as the neuroinflammation, or generalized inflammation, persists, well after 72 hours post-surgery^13,14^.

## Methods

### Study sample

The study was approved by the Institutional Review Board at the University of Pennsylvania (#815686). The study was performed by the ethical standards in the 1964 Declaration of Helsinki and its later amendments. All participants in the study provided written informed consent.

The study sample consisted of serial collection of blood samples before surgery (t_0_), and 24 hours (t_+24h_), 7 days (t_+7d_), and 3 months (t_+3m_) after the cardiac surgery. All patients were scheduled for elective heart surgery at two university hospitals in the northeast region of the United States. The average case volume exceeds 500 per year in either location. The demographic and entry characteristics are presented in Table 1. Five different surgeons participated in the study. Due to the high protocolization of pre-, intra- and post-operative care, the samples were relatively homogenous in terms of the use of non-steroidal inflammatory drugs, statins, and peri-surgical steroid dosing (Table 1).

**Table 1:** Clinical characteristics of the study sample.

Patient Characteristics (N = 30)	
Age, mean(SD), years	67.9 (8.85)
Sex - Male no (% of total).	21.0 (70%)
BMI mean(SD) [kg/m²]	26.9 (5.3)
**Anesthesia & Surgery Data**	
Duration of anesthesia; mean(SD) [min]	389.3 ± 89.07
Duration of surgery; mean(SD) [min]	272.1 ± 83.2
Duration of CBP, mean(SD) [min]	123.5 ± 56.5
Duration of X-clamp, mean(SD) [min]	80.2 ± 44.4
Coronary artery bypass surgery no. (% of total).	10 (33%)
Mitral valvuloplasty no. (%ana of total).	8 (26.7%)
Aortic valve replacement no. (% of total).	7 (23.3%)
Aortic aneurysm repair no. (% of total).	2 (6.7%)
Others, no (% of total).	3 (10%)
**Opioid/Sedative Usage**	
*During Surgery*	
Morphine Equivalents, mean(SD) [mg]	120.3 ± 42.6
Midazolam, mean(SD) [mg]	4.4 ± 2.1
Corticosteroid Administration (% of all cases)	10
*In 24* *h post-surgery*	
Morphine Equivalents, mean(SD) [mg]	32.4 ± 38.8
Midazolam, mean(SD) [mg]	1.2 ± 4.3
Aspirin Administration (% of all cases)	60
Ketorolac Administration (% of all cases)	3.3
**Transfusions**	
*During surgery*	
Packed Red Blood Cells, mean (CI95%) [ml]	0 (0;1200)
Fresh Frozen Plasma, mean (CI95%) [ml]	0 (0;2250)
Platelets, mean (CI95%) [ml]	0 (0;1032)
*In 24* *h post-surgery*	
Packed Red Blood Cells, mean; (CI95%) [ml]	0 (0;600)
Fresh Frozen Plasma, mean; (CI95%) [ml]	0 (0;900)
Platelets, mean; (CI95%) [ml]	0 (0;900)
Total Crystalloid during surgery[ml]	2500 (400;5200)
**ICU stay**	
APACHE score at 1 h, mean(SD)	20.5 (4.8)
APACHE score at 24 h, mean(SD)	12.0 (4.9)
APACHE score at 48 h, mean(SD)	12.1 (5.7)
**Comorbidities**	
Acute Coronary Syndrome	9 (30.0%)
Chronic heart failure	5 (16.7%)
Connective tissue disease (non-active)	5 (16.7%)
Peripheral vascular disease	3 (10.0%)
Cerebrovascular disease	4 (13.3%)
Type 2 diabetes	1 (3.3%)
Liver disease	0 (0%)
AIDS	0 (0%)
COPD	1 (3.3%)
Any tumor (last five years)	1 (3.3%)
Renal failure (moderate-severe)	5 (16.7%)

We approached a total of 51 patients. 37 patients agreed to 3 months of follow up. A total of 30 patients were finally included in the analysis due to sample availability (all four time points were collected). The sample size was based on prior comparable studies^14,39^.

### Measurement of neurodegeneration and inflammatory markers

Blood samples were collected in EDTA tubes (BD Biosciences, San Jose, CA), at the times specified above. The samples were centrifuged within 30 minutes of collection to separate serum from cellular components. Serum was subsequently stored at −80 °C until measurement was performed.

Plasma concentration of neurodegeneration markers was measured in the serum using the SiMoA neurology 4-plex assay which measures GFAP, UCH-L1, total tau, and NF-L in a serum sample simultaneously. The lower limits of detection of the assay for GFAP, UCH-L1, total tau, and NF-L are 0.221 pg/ml, 1.74 pg/ml, 0.024 pg/ml, and 0.104 pg/ml, respectively. The inter-lot and inter-instrument coefficient of variation (CV) for each of the proteins were <5%. All samples were run in duplicates. The serum level of CRP, serum amyloid protein α, α-2-macroglobulin, and haptoglobin were measured using a multiplex kit (Millipore, Burlington MA). The samples were run on Flex 3D (Bio-Rad; Hercules CA). The detection limit is specified by the manufacturer.

### Measurement of monocyte activation

MOs were separated as described previously and stimulated for 18 hours with lipopolysaccharide (LPS; [50 ng/ml]; Enzo Biological; Farmingdale NY)^39^. The TNFα (Biolegend, San Diego CA) and M-CSF (Thermofisher, New York NY) levels in supernatants were measured using the ELISA technique as described by the manufacturers.

### Statistical analysis

A preliminary power calculation was conducted based on prior studies investigating the changes in serum level of neuronal damage biomarkers and prolonged MO activation^14,39^. We needed to collect data on 29 patients in order to achieve α of 0.05 and power of 0.85 with ~25% increase in serum tau level as compared to pre-surgical level assuming a bilateral null hypothesis.

Blood samples were taken at four different time points (baseline (t_0_), 24 hours (t_+24h_), 7 days (t_+7d_), and 3 months (t_+3m_)) from patients undergoing heart surgery with the application of cardiopulmonary bypass. Data were compared to the pre-CPB values of the same patient (t_0_). For some analysis, we used 25% elevation in serum level of neurodegeneration marker based on prior analysis and perception of significance^14,33–35,39^.

Mean (X) and standard deviation (SD) were used to present the parametric data while median (M_e_) and 75% upper and 25% lower quartile (IQ) were chosen for non-parametric data. The parametric nature of the data was confirmed by Levene and Shapiro-Wilk tests. *t*-tests for repetitive samples and Wilcoxon matched pair tests were conducted depending on the characteristics of the data. ANOVA or Kendall’s test was conducted for multiple group comparisons with a Bonferroni statistic for post-hoc analysis. Most comparisons were done through longitudinal analysis using patient pre-CPB values (t_0_) as the baseline unless otherwise specified. The data was flagged as significant if the one-tailed hypothesis test resulted in p < 0.05 on both ends of confidence intervals. Statistica v11.0 (Statistica, Tulsa, OK) was used for data analysis.

## Results

### Tau protein is significantly and persistently elevated after cardiac surgery with the application of cardiopulmonary bypass

**Figure 1:**
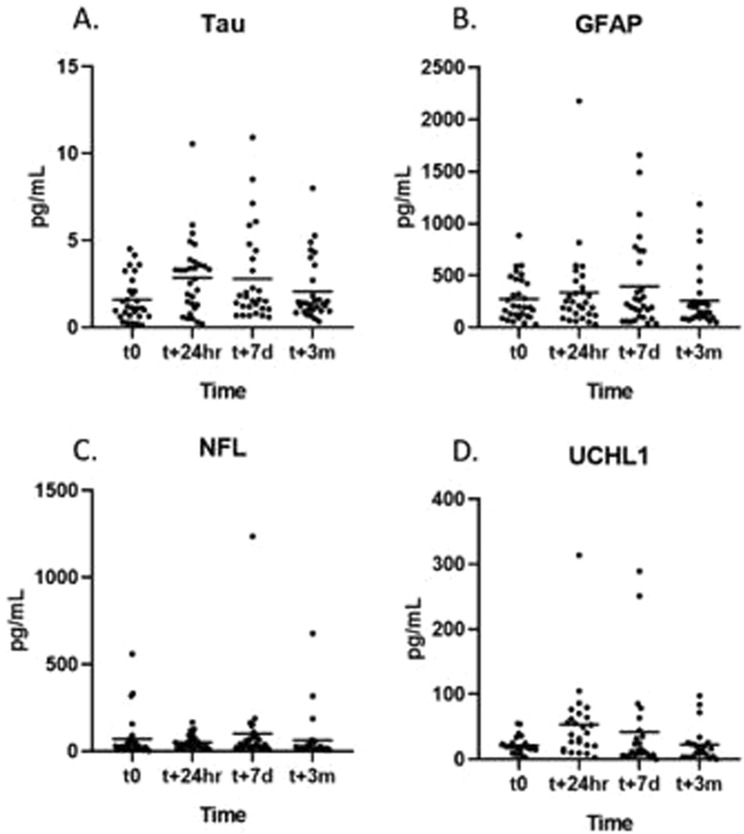
Distribution of biomarker levels at each time point.

**Figure 2:**
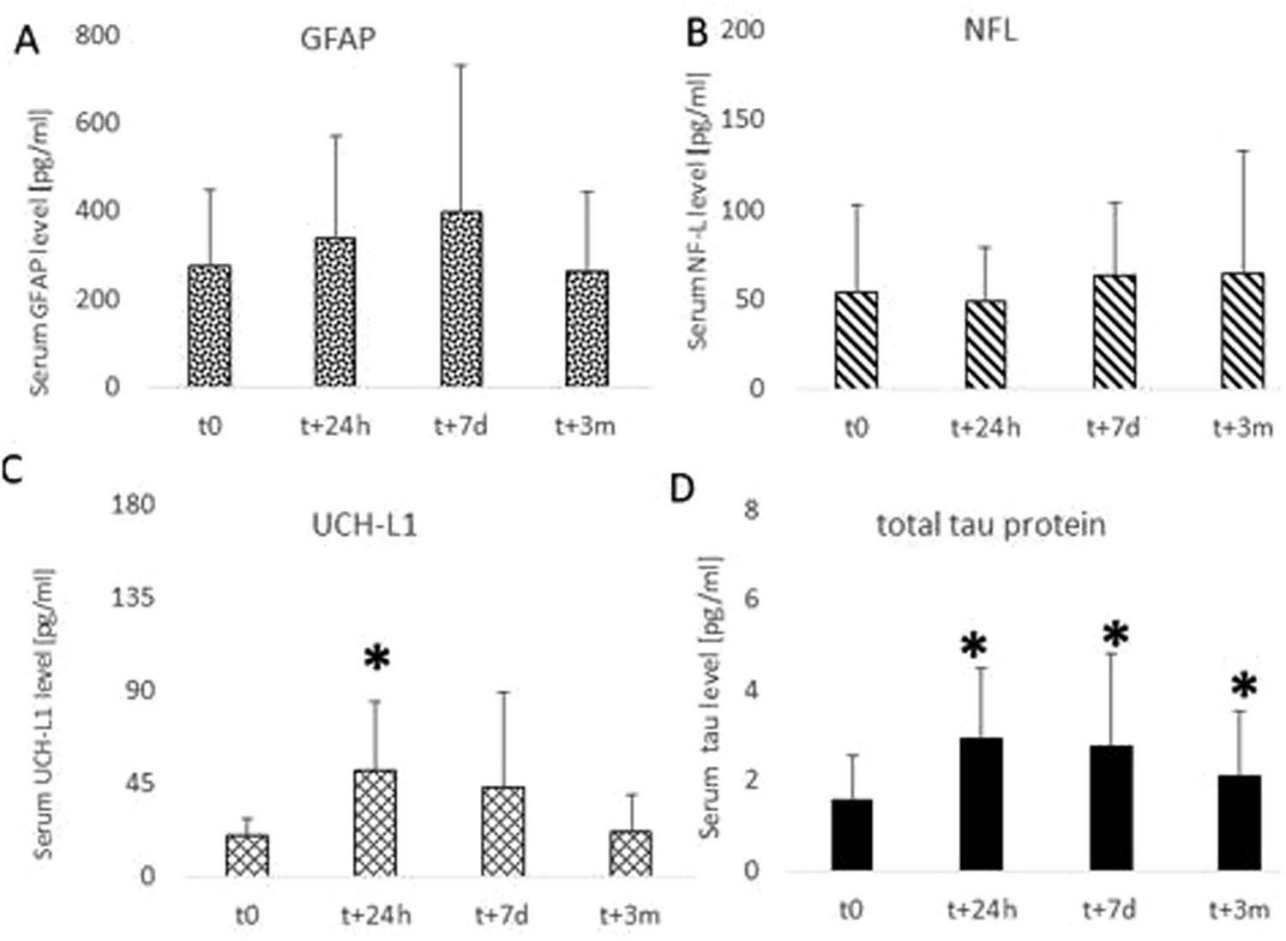
Changes in neurodegeneration markers level after cardiac surgery with involvement of cardiopulmonary bypass (CPB). The level of NF-L and GFAP was highly variable (**A**,**B**). UCH-L1 showed significant increase in serum levels at 24 hours after surgery while tau protein levels remained elevated even 3 months after CPB (**C**,**D**). *Denotes significance level below 0.05.

**Figure 3:**
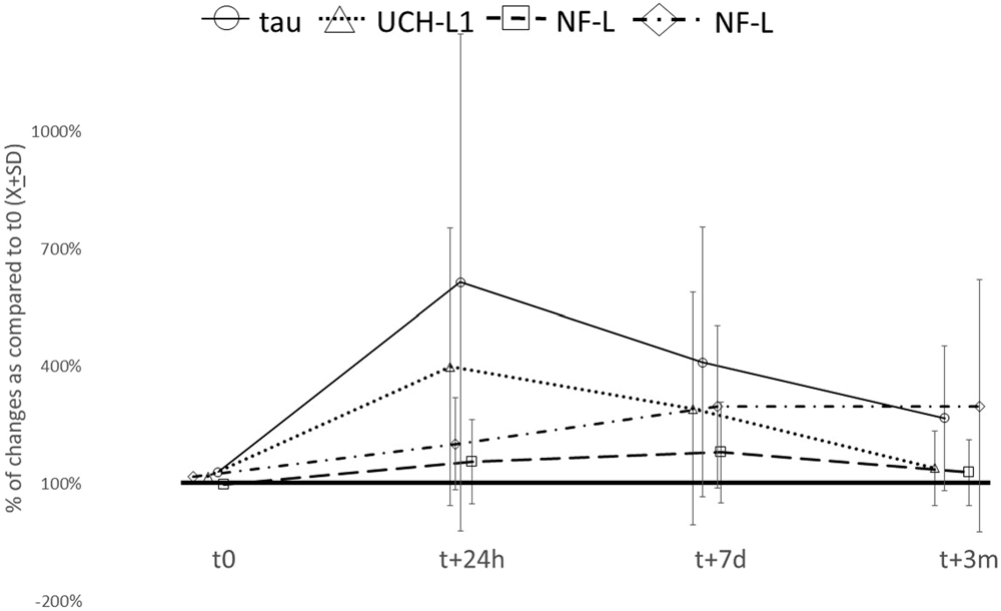
The relative changes in serum level of neurodegeneration markers showed different time characteristics for each marker after cardiac surgery.

We analyzed the post-CPB dynamic of serum levels of four markers of neurological injury. The distribution of serum levels of each neurodegeneration marker varied significantly at each time point (Fig. 1). Neither serum GFAP nor NF-L had a significant increase after surgery over time, though significant variability existed in the case of NF-L serum levels (Fig. 2A,B). UCH-L1 was elevated at 24 hours (UCH-L1_t0_ = 21.6 ± 13.8 CI95% 10.4;18.6 *vs* UCH-L1_t24h_ = 53.5 ± 60.9 CI95% 47.6;84.8; p = 0.00055) but normalized at the remaining points (Fig. 2C). Initial serum level of tau was low (tau_t0_ = 1.6 ± 1.26 CI95% 1.0;1.7) but significantly increased at 24 hours after surgery (tau_t24h_ = 2.9 ± 2.14 CI95% 1.7;2.9; p = 0.0015) and remained elevated at both 7 days (tau_t7d_ = 2.8 ± 2.68 CI95% 2.14;3.65; p = 0.0017) and 3 months (tau_t7d_ = 2.1 ± 1.39 CI95% 1.43;2.44; p = 0.036) (Fig. 2D). The different temporal characteristics of the markers can be appreciated more when a relative change to pre-CPB levels is visualized (Fig. 3). Among the individuals with an increase of serum tau protein over 25% from pre-CPB baseline at 3 months after surgery, only UCH-L1 was borderline co-elevated (tau_[high]_ = 31.2 ± 32.2 vs. tau_[low]_ = 10.86 ± 8.24; t = 1.93; p = 0.066) but the levels were highly varied.

The pre-CPB level of respective neurological markers correlated with increases at 3 months for GFAP (*r* = 0.40; *p* = 0.034), UCH-L1 (*r* = 0.49; *p* = 0.028) and tau protein (*r* = 0.49; *p* = 0.008) but not NF-L. Tau levels at 24 hours correlated with APACHE admission score (*r* = 0.41; *p* = 0.031), but other markers did not. The serum levels of neurological markers of injury did not significantly correlate with the Charlson Comorbidity Index, age, gender, duration of anesthesia, surgical attending, cross-clamp and surgery length, or length of ICU or hospital stay (data not shown).

**Figure 4:**
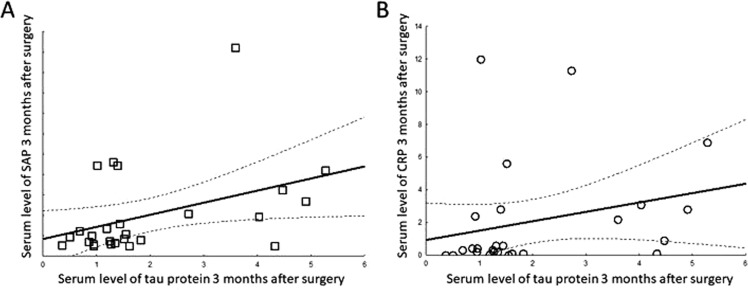
SAP and CRP (to some extent) correlateed with post-CPB elevation of tau protein. Regression line is visualized by a continuous line while 95% confidence intervals are visualized by broken lines.

We correlated the circulating characteristics of MO with changes in tau protein at 3 months. Production of both cytokines was elevated at 3 months in response to LPS as compared to pre-CPB levels (data not shown). *In vitro* production of M-CSF in response to LPS correlated highly (*r* = 0.58; *p* = 0.04) with persistent elevation of serum tau levels. Production of TNFα in response to LPS showed non-significant correlations (*r* = 0.27; *p* = ns). Serum tau protein had a low but significant correlation with SAP (*r* = 0.40; *p* = 0.041; Fig. 4A) and CRP (*r* = 0.47; *p* = 0.01; Fig. 4B) but not α-2-macroglobulin (*r* = 0.072; *p* = 0.709; data not shown).

## Discussion

This study demonstrated the persistence of elevations in serum tau levels at 24 hours, 7 days, and 3 months after cardiac surgery involving cardiopulmonary bypass. Elevation in S100 has been observed up to 72 hours post cardiac surgery while serum NF-L was elevated 48 hours after non-cardiac surgery^13,14,33^. NF-L was reported to be elevated up to 12 days after traumatic brain injury^40^. The increase in serum tau protein correlated with some acute phase proteins and production of M-CSF by isolated MO. None of the anesthesia, surgical, or recovery factors were related to the peak levels of studied neurological markers.

Here, we demonstrated that serum tau remained elevated beyond 7 days after cardiac surgery. The degree of elevation in tau protein at three months correlated only with the pre-surgical tau levels. This observation correlates well with other studies suggesting that only pre-existing brain vulnerability or degree of neuronal insult is related to POCD^3,4,7,41,42^. Other markers showed different time dynamics with UCH-L1 peaking at 24 hours. The sequential nature of the changes in serum levels of UCH-L1 and tau suggests that their leak into the bloodstream is a combination of a post-CPB increase in their tissue expression and ongoing damage to the neuronal tissues. NF-L release has been demonstrated previously to be elevated for prolonged periods of time^14,34^. We reported NF-L levels conservatively as non-significant due to the high variability in values, despite non-parametric analysis being borderline statistically significant.

GFAP, NF-L, and UCH-L1 are critical to withstand stress. Their cellular expression are reflective of post-DAMP or PAMP exposure while their release is reflective of ongoing tissue necrosis^32,33,35,36^. In contrast, tau protein is predominantly seen in patients with ongoing neurological disorders, suggesting that its release is a function of pre-surgical degenerative processes which is exacerbated by the stress related to cardiac surgery^1,20^. Neuroinflammation has been frequently suggested as CPB-initiated, but once started it may become a self-sustained process^22,24^. Microglia are linked to post-surgical neuro-inflammation^22,23,43^. Though we did not study the microglia directly, we observed increased levels of M-CSF in peripheral blood monocytes and serum levels of SAP for over a 3-month period. Similar persistence in microglia activation has been reported after heart surgery and the inflammatory process^24,39^. Considering high homology between peripheral blood MO and microglia, observed activation of peripheral MO reflect persistent microglia priming by CPB^27,29^. Also, M-CSF has been linked to a release of tau protein and cognitive decline^28^. However, it is also possible that the release of neurodegeneration markers triggered mononuclear cell activation and their migration inside the CNS^25,26^.

Our study could not account for some variables. Postoperative cardiology care may impact the recovery of patients. Statin and ASA in particular are related to modulation of the inflammatory responses^33^. On the other hand, cardiac care after surgery within our health system is highly standardized. Though we collected several anesthetic, operator and surgical factors; we did not quantify the frequency and degree of intraoperative hypotension, hypoxemia, hyperoxia, and acidosis^15,20,44–46^. However, the definition of any of these complications is ambiguous and confusing at best^47^. Our study was not intended to formally investigate neurocognitive outcomes and link them to the elevation of the neurodegeneration markers. From all studied individuals, only three were in a rehabilitation center at 28 days after surgery, and all of them were home at 3 months. This suggests that they attained a functional level of recovery comparable to that before surgery. It is likely that post-CPB injury takes a significant amount of time to develop while our window of observation (3 months) was too short or too subtle to detect^2,5–7,44^.

In summary, our study showed the persistent elevation of serum tau protein after heart surgery with the implementation of cardiopulmonary bypass. The increase in serum tau protein correlated with production of M-CSF in response to LPS, a potential surrogate of microglial priming.

## Conclusion

Our study aimed at understanding the dynamics of neuroinflammatory markers among individuals undergoing cardiac surgery with the application of CPB. Most importantly, our study demonstrated a delay in the peak time of neurodegeneration markers that could be a factor of CPB implementation. While the rise in neuronal injury markers has been well documented in prior studies, its elevation was not documented past 72 hours post-insult. This also calls for further investigations which could potentially involve a longitudinal follow up of individuals undergoing cardiac surgery with CPB.

## Supplementary information

Supplementary Figure 1.
