# Supplementary material for: Observational study of long-term persistent elevation of neurodegeneration markers after cardiac surgery
Source: Sci Rep. 2019 May 9;9:7177. doi: 10.1038/s41598-019-42351-2 (PMC6509119; doi:10.1038/s41598-019-42351-2)
Supplement: Supplementary file 1 — Supplementary Figure 1. [file 41598_2019_42351_MOESM1_ESM.pdf]

Observational study of long-term persistent elevation of neurodegeneration markers after cardiac surgery.

Matthew DiMeglio MBA[1,4], William Furey MBA[1,4], Jihane Hajj CRNP[2], Jordan Lindekens BSN[3], Saumil Patel MD[4], Michael Acker MD[5], Joseph Bavaria MD[5], Wilson Y Szeto MD[5], Pavan Atluri MD[5], Margalit Haber PhD[6], Ramon Diaz-Arrastia MD, PhD[6], Krzysztof Laudanski MD, PhD, MA, FCCM[4*]

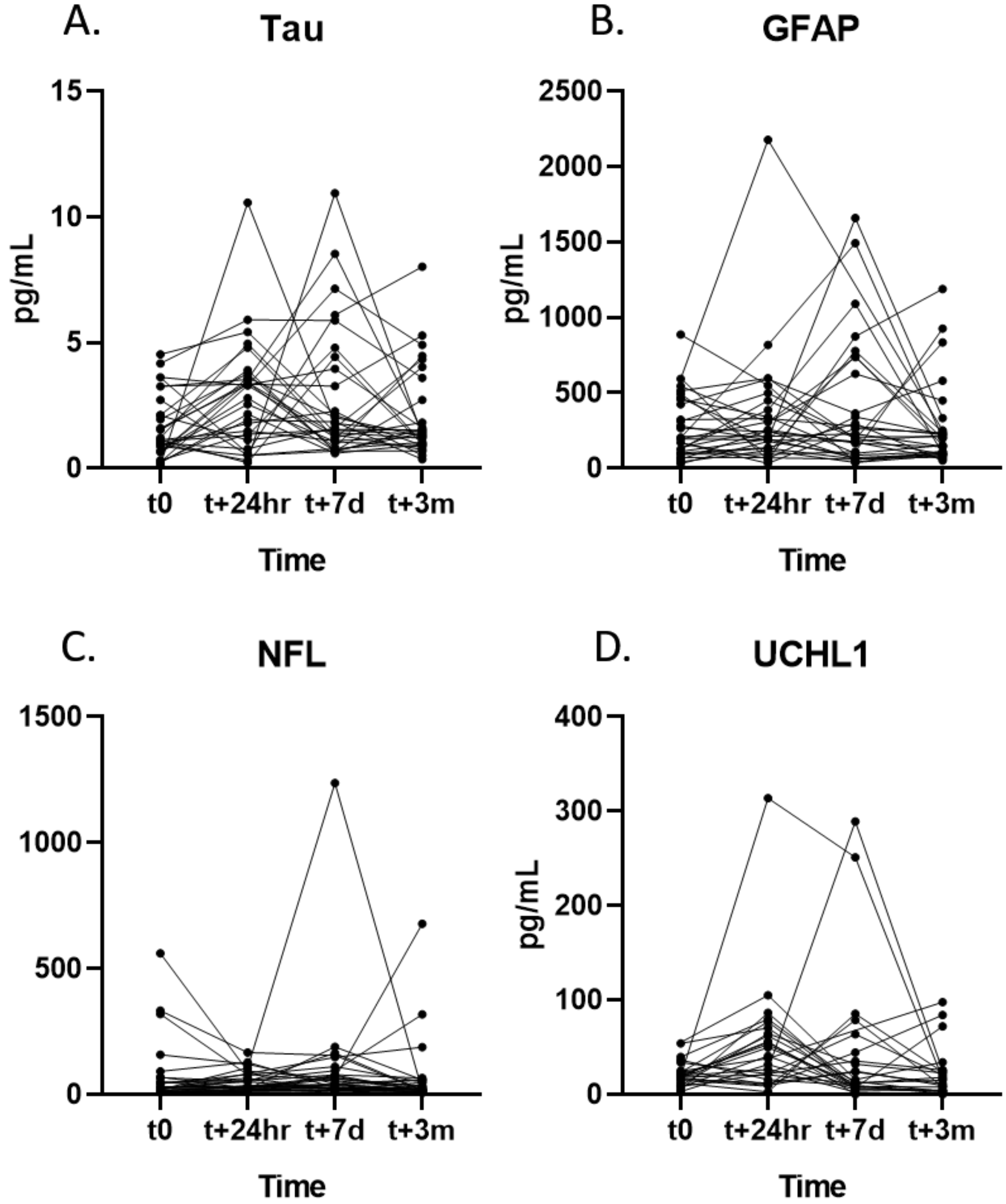

Supplemental Figure 1. Spaghetti plot of biomarker values for each patient at all time points.
